# Clinical research updates

**DOI:** 10.1111/camh.12769

**Published:** 2025-05-15

**Authors:** Marinos Kyriakopoulos, Eleni Vrigkou, Markos Kallinikos, Zinovia Maridaki

**Affiliations:** ^1^ Department of Psychiatry National and Kapodistrian University of Athens Athens Greece; ^2^ Department of Child and Adolescent Psychiatry Institute of Psychiatry Psychology and Neuroscience, King's College London London UK; ^3^ South London and Maudsley NHS Foundation Trust London UK

## The social skills of autistic boys in preschool: the contributions of their dyadic and triadic interactions with their parents

Eleni Vrigkou

One of the key developmental tasks in early childhood is to adapt to the social environment of preschool. The social and communication challenges of autistic children can make this process more demanding compared to typically developing (TD) children. Empirical data, mainly from TD children, suggest that the social skills they acquire through parent–child interactions can be associated with their ability to form adequate social abilities in preschool, but a question remains of whether the same could apply to autistic children as well.

Oppenheim et al. (2025) conducted a study in autistic preschooler boys to assess the boy's engagement in dyadic (mother–child and father‐child) and triadic (mother–father‐child) interactions and its association with their social skills in preschool, while controlling for the severity of their autism symptomatology and their IQ. A particular focus was added on the importance of triadic interactions over and above their dyadic ones. The study was conducted in 2 time‐points (T1 and T2), 12 months apart. Seventy‐five boys, 29–68 months old, and their parents participated in T1 time‐point and 68 in T2. T1 included 4 laboratory and preschool visits, where dyadic and triadic interactions observations, autism and cognitive functioning assessments, and social skills assessments were conducted. T2 included only one preschool observation visit for social skills evaluations. The boys' behavior in the dyadic interactions was assessed using the Child Responsiveness to Child Involvement with Parent scales in free and social play. Triadic interactions were observed in the Lausanne Triadic Play procedure. Their social skills were assessed using the Social Skills Q‐sort (SSQ) completed by teachers and observers and the Social Responsiveness Scale (SRS) completed by teachers.

The authors found that the boys' dyadic engagement predicted the observers' SSQ in T1 and T2, and the teachers SRS in T1. Their triadic engagement did not explain additional variance in any social skill measure in T1 but accounted for additional variance in all social skills measures in T2. Their findings suggest that dyadic engagements are important for the boys' social skills in preschool, and that triadic engagements seem to be particularly significant, above and beyond dyadic contexts, especially over time. The contributions of the dyadic and triadic engagements were not different for children with different levels of social abilities at T1.

The authors identified two strengths of the study: the longitudinal design that allowed the assessment of the boys' social abilities over time and the fact that all children were in the autism group. Study limitations were also identified, including the small added variance of the triadic engagements over the dyadic ones in some cases, the fact that possible mechanisms that connect children's family interactions with their interactions in social environments beyond the family were not explored, and the inclusion of only boys as study participants. In conclusion, the findings of this study support the hypothesis that autistic boys' engagements in dyadic and triadic contexts can be transferred to their preschool social environment, with triadic engagements playing a potentially significant role.

Oppenheim, D., Mottes‐Peleg, M., Hamburger, L., Slonim, M., Maccabi, Y., & Yirmiya, N. (2025). The social skills of autistic boys in preschool: The contributions of their dyadic and triadic interactions with their parents. *Journal of Child Psychology and Psychiatry*, 66, 322–332.

## Callous‐unemotional traits, cognitive functioning, and externalizing problems in children

Markos Kallinikos

Several lines of evidence suggest that externalizing problems in children and adolescents might be influenced by the presence and severity of callous‐unemotional (CU) traits and cognitive difficulties.

Murtha et al. (2025) explored, through the data of the Adolescent Brain Cognitive Development (ABCD) study, associations between CU traits, cognitive functioning and parent‐reported symptoms of conduct disorder (CD), oppositional defiant disorder (ODD), and attention deficit hyperactivity disorder (ADHD) on a cross‐sectional level. Longitudinal outcomes were child‐reported overt and relational aggression. By applying propensity matching, two groups of children were formed, one with CU traits, and one without CU traits, while controlling for sociodemographic variables. Data were collected using behavioral, child and parent reports. Parent Longitudinal Demographic Questionnaire, 1 Item from the Child Behavior Checklist and 3 items from the Strengths and Difficulties Questionnaire, and the NIH Cognition Battery Toolbox were employed to assess sociodemographic factors, CU traits and cognitive abilities respectively. Analysis was conducted using regression modeling in Mplus and R Package ‘MatchIt’. Cross‐sectional outcomes indicated possible associations between dimensionally‐assessed CU and ADHD, and CD scores. All three cognitive domains evaluated (general ability, executive function, and learning and memory) were significantly negatively associated with ADHD and CD, and executive function and learning and memory were significantly negatively related to ODD. Lower general cognitive ability predicted higher levels of parent‐reported CU traits in the moderation models. More child‐reported overt and relational aggression was observed in dimensionally assessed CU traits in the longitudinal design. General cognitive ability and learning and memory scores were positively associated with relational aggression, while executive function and learning and memory scores were negatively associated with overt aggression. Higher ratings in CU traits also appeared more frequently together with ADHD and ODD symptoms. Learning and memory were negatively associated with ODD and CD symptoms. CU group membership was related to both forms of aggression in the longitudinal model with cognition not appearing to be a significant moderator.

Limitations of the study included lack of control for sex, household income, and parental education, the CU traits measure consisting of just four items, and the ABCD community sample having low prevalence rates of severe externalizing psychopathology. The study highlighted that CU traits are a robust and unique risk factor for externalizing psychopathology and need to be specifically evaluated and targeted through specific interventions.

Murtha, K., Perlstein, S., Paz, Y., Seidlitz, J., Raine, A., Hawes, S., … & Waller, R. (2025). Callous‐unemotional traits, cognitive functioning, and externalizing problems in a propensity‐matched sample from the ABCD study. *Journal of Child Psychology and Psychiatry, and Allied Disciplines*, 66, 333–349.

## Efficacy and safety of antipsychotics vs antiepileptics or lithium for acute mania in children and adolescents

Zinovia Maridaki

Bipolar disorder (BD) is one of the most debilitating psychiatric disorders. BD before the age of 18 years is associated with a more severe course of illness and worse long‐term clinical outcomes and also presents multiple diagnostic and therapeutic challenges. The guidelines for the treatment of BD in this age group recommend the use of second‐generation antipsychotics (SGAs) and mood stabilizers (MSs) but their relative efficacy and safety are unclear.

Vita et al. (2025) conducted a systematic review and network meta‐analysis (NMA) to investigate the safety and efficacy of SGAs and MSs in patients under the age of 18 years diagnosed with a manic or mixed episode of Bipolar I Disorder (BD‐I). They used PRISMA guidelines and included 18 randomized clinical trials, with a total of 2,844 patients (52% male, 70% white) aged 3–18 years old (mean age 11.74 ± 2.5 years). At baseline, 53.2% of the patients were in a mixed episode, and 46.8% were in a manic episode. 53.3% of the patients had comorbid attention deficit hyperactivity disorder (ADHD), whereas 43.6% had comorbid oppositional defiant disorder (ODD). Medication efficacy was assessed by the mean change in manic symptomatology and the mania response at the study endpoint, as well as the pooled analysis by drug class. It identified that, pooled together, SGAs including risperidone, olanzapine, aripiprazole, quetiapine, asenapine, and ziprasidone were more efficacious than both placebo and MSs. Risperidone was more efficacious in reducing manic symptomatology compared to most other treatments, with the exception of olanzapine and topiramate, yet with low/very low confidence. Among the MSs, including lithium, oxcarbazepine, topiramate, and valproate, lithium was the only medication that outperformed placebo. In terms of functional improvements, SGAs were also more efficacious than MSs and placebo. There was no difference in all‐cause discontinuation compared to placebo in all treatments. However, oxcarbazepine, ziprasidone, and lithium were associated with significantly more dropouts due to adverse events. SGAs were linked to sedation and significant metabolic adverse effects, such as weight gain, increased serum glucose, cholesterol, and prolactin levels. Risperidone was associated with hyperprolactinemia and increased glucose.

The authors hypothesized that the apparent superiority of risperidone in comparison to other SGAs and MSs could be linked to the alleviation of symptoms of an underlying or comorbid neurodevelopmental disorder, a suggestion also supported by the literature. Risperidone did not seem to be superior compared to other SGAs when participants younger than 12 years old were excluded from the analysis. In addition, the high rates of ODD and ADHD in this population raise the possibility of inaccurate BD diagnoses, especially among the prepubertal participants. Limitations of the NMA include the small number of studies, the limited direct comparisons between specific drugs (head‐to‐head trials), limited generalizability, statistical limitations of the pooled analysis, and, lastly, the short duration of trials (mean follow‐up of 5.4 weeks).

This NMA highlights that SGAs may be more efficacious than MSs in the acute treatment phase of BD‐I in children and adolescents with moderate evidence but are associated with significant side effects requiring long‐term monitoring.

Vita, G., Nöhles, V.B., Ostuzzi, G., Barbui, C., Tedeschi, F., Heuer, F.H., … & Correll, C.U. (2025). Systematic review and network meta‐analysis: Efficacy and safety of antipsychotics vs antiepileptics or lithium for acute mania in children and adolescents. *Journal of the American Academy of Child and Adolescent Psychiatry*, 64, 143–157.

## Conflict of interest statement

M.K. is the CAMH Associate Editor for Clinical Research Updates. The editor thanks the contributors for this issue's Clinical Research Updates. The editor has declared that he has no competing or potential conflicts of interest.

## Ethics statement

No ethical approval was required for these updates.

## Data Availability

Data sharing is not applicable to this article as no new data were created or analyzed.

